# Remifentanil-induced penile erection in elderly patients undergoing urethroscopic surgery

**DOI:** 10.1186/s40981-021-00487-z

**Published:** 2021-11-29

**Authors:** Hiroaki Maru, Shinju Obara, Satoki Inoue

**Affiliations:** 1 Department of Anesthesiology, Fujita General Hospital, 14 Tsukanome Sanbongi, Kunimi, Fukushima 969-1793 Japan; 2 Department of Anesthesiology, Fukushima Medical University, 1 Hikarigaoka, Fukushima, Fukushima 960-1295 Japan

**To the Editor**

Remifentanil, an ultra-short-acting opioid, blunts sympathetic responses to anesthetic or surgical stimuli and may affect the balance between sympathetic and parasympathetic nerves. Parasympathetic tone is generally predominant in young people and remifentanil-induced penile erection is observed in male children and young adults, probably resulting from an increased activity of parasympathetic nerve relative to sympathetic nerve [1, 2]. Moreover, it seems that this phenomenon can be explained by the theory that the amount of parasympathetic activity in younger patients would increase by remifentanil, compared to the amount of sympathetic activity [2]. However, we have occasionally observed penile erection in elderly patients in our institution, especially just before urethroscopic procedures, after anesthesia induction with remifentanil. At that time, general anesthesia was usually induced with propofol 1-2 mg/kg, fentanyl 2μg/kg, remifentanil 0.4-0.5 μg/kg/min, and vecuronium 0.15mg/kg was used for tracheal intubation. Anesthesia was maintained 1.5% sevoflurane in 40% oxygen and remifentanil 0.2-0.3 μg/kg/min after induction. We suspect that remifentanil induces penile erection, even in elderly patients, because the series of events leading to penile erection cease immediately after stopping infusion of remifentanil (in approximately 5 min). In addition, we first encountered this phenomenon at around the same time that remifentanil was made clinically available in Japan. There had been at least no less than 5 cases by the time we were convinced of this phenomenon. Of those, some patients had intracorporeal injection of phenylephrine used to treat penile erection instead of stopping infusion of remifentanil before we noticed the association between remifentanil and penile erection. The following mechanisms can be proposed for remifentanil-induced penile erection in elderly patients undergoing urethroscopic procedures. First, parasympathetic activity relative to sympathetic activity would be increased by remifentanil even in elderly patients. Second, remifentanil induces vasodilation, partly via nitric oxide in an endothelium-dependent mechanism [3]. Lastly, external genital preparation before urethroscopic procedures is likely to cause enough physical stimuli to contribute to the induction of penile erection. Because it is extremely difficult and dangerous to perform urethroscopic procedures while the patient is experiencing penile erection, we more likely to notice this phenomenon in elderly patients undergoing urethroscopic procedures although this phenomenon may occur in other situations.

To the best of our knowledge, this is the first report of remifentanil-induced penile erection in elderly patients undergoing urethroscopic procedures. Because urethroscopic surgery is usually performed under spinal anesthesia without the use of remifentanil and penile election is rarely seen under spinal anesthesia [4], intraoperative penile erection by remifentanil is hardly detectable. However, it should be understood that this phenomenon can occur in not only male children and young adults, but also elderly patients. When encountering this situation, we recommend trying to stop the infusion of remifentanil. The exact frequency of this phenomenon is still unclear, because we have not used remifentanil for elderly patients undergoing urethroscopic procedures since we began to suspect its potential occurrence. Since then, we still have encountered a few cases of penile erection in patients undergoing urethroscopic procedures. However, it has been reported that penile erection under spinal or general anesthesia at the time of urethroscopic surgery occurs at a reported incidence of 0.1–2.4% [4]. However, by our estimation, it does not feel like that the rate of penile erection with remifentanil is not lower than the previously reported ones.

## Data Availability

Not applicable
